# PANGEN: an online platform for the comparison and creation of diagnostic gene panels

**DOI:** 10.1093/database/baae065

**Published:** 2024-07-23

**Authors:** Ofer Isakov, Dina Marek-Yagel, Rotem Greenberg, Michal Naftali, Shay Ben-Shachar

**Affiliations:** Raphael Recanati Genetic Institute, Rabin Medical Center-Beilinson Hospital, Zeev Jabotinsky 39, Petach Tikva 4941492, Israel; Clalit Research Institute, Clalit Health Services, Tuval 40, Ramat Gan 5252247, Israel; The Ivan and Francesca Berkowitz Family Living Laboratory Collaboration, Harvard Medical School and Clalit Research Institute, 10 Shattuck Street, Suite 514, Boston, MA 02115, USA; Faculty of Medicine, Tel Aviv University, Klachkin 35, Tel Aviv 6997801, Israel; Clalit Research Institute, Clalit Health Services, Tuval 40, Ramat Gan 5252247, Israel; Clalit Research Institute, Clalit Health Services, Tuval 40, Ramat Gan 5252247, Israel; Clalit Research Institute, Clalit Health Services, Tuval 40, Ramat Gan 5252247, Israel; Clalit Research Institute, Clalit Health Services, Tuval 40, Ramat Gan 5252247, Israel; The Ivan and Francesca Berkowitz Family Living Laboratory Collaboration, Harvard Medical School and Clalit Research Institute, 10 Shattuck Street, Suite 514, Boston, MA 02115, USA; Faculty of Medicine, Tel Aviv University, Klachkin 35, Tel Aviv 6997801, Israel

## Abstract

Targeted gene panel sequencing is used to limit the search for causative genetic variants solely to genes with an established association with the phenotype. The design of gene panels is challenging due to the lack of consensus regarding phenotypic associations for some genes, which results in high variation in gene composition for the same panel offered by different laboratories. We developed *PANGEN*, a platform that provides a centralized resource for gene panel information, with the ability to compare and generate new intelligent diagnostic panels. Gene–phenotype associations were collected from 12 public and commercial sources (Blueprint, Cegat, Centogene, ClinGen, Fulgent, GeneDx, Health in Code, Human Phenotype Ontology, Invitae, PanelApp, Prevention genetics, and Pronto diagnostics). Gene–phenotype associations are categorized into tiers according to categories derived from the original source panel. Pairwise panel similarity was calculated by dividing the number of common genes by the total number of genes in both panels. Regions with extreme guanine-cytosine (GC) content were collected from the Genome in a Bottle stratifications dataset, and putative genomic duplications were retrieved from the University of Santa Cruz database. Overall, 1533 panels, 9759 phenotypes, and 6979 genes were collected. The platform provides an interface to (i) explore and compare collected panels, (ii) find similar panels, (iii) identify genes with high GC content or duplication levels, (iv) generate gene panels by combining panels from various sources, and (v) stratify a generated panel into genes with a strong phenotype association (‘core’) and those with a weaker association (‘extended’). The presented platform represents a unique resource for gene panel exploration and comparison that facilitates the generation of tailored diagnostic panels through a public online web server.

**Database URL**: https://c-gc.shinyapps.io/PANGEN/

## Background

Targeted gene panel sequencing is used to limit the search for causative genetic variants to genes associated with specific phenotypes, thereby maximizing target coverage and avoiding incidental findings [1, 2]. Targeted panels may be defined prior to sequencing, during library preparation steps, or as virtual gene panels based on whole exome/genome sequencing panels (WES/WGS). Such virtual panels are becoming increasingly common, as they enable dynamic panel design considering various degrees of gene–phenotype association [2]. The process of gene panel design presents a challenge due to the lack of consensus across the scientific community and commercial sources regarding gene–phenotype associations. Collaborative tools, such as PanelApp, facilitate gene panel design based on expert review and include mostly genes with a strong association to the phenotype [3]. While this approach controls the amount of identified variants of unknown significance (VUS), it limits the sensitivity of the panel to identify causative variants in genes with weaker phenotypic association. Consequently, there is considerable variability in the composition of gene panels that address the same clinical condition, making it challenging to determine the most appropriate panel for a given case. This variability can lead to inefficiencies in genetic testing and potentially missed diagnoses, thereby underscoring the need for a standardized and centralized resource. A comprehensive assessment of the spectrum of available panels for a given phenotype facilitates the inclusion of genes that could otherwise be missed while considering the origin of each gene in the panel. Careful virtual gene panel design is critical since these panels have been shown to result in an increase in reported VUS compared to WES, due in part to differences in reporting practices, and therefore consideration should be taken when deciding on their gene content [4]. This necessitates performing several labour-intensive tasks, such as retrieving gene content from various providers for relevant panels, consolidating and differentiating these panels, and prioritizing genes based on their source. In light of these challenges, we have developed PANGEN (targeted gene **PAN**el **GEN**erator), a unique platform that represents a unified, comprehensive resource for gene panel information. Our platform not only aggregates data from a diverse set of public and commercial sources but also offers advanced tools for panel comparison and customization.

## Methods

### Data collection and compilation

An extensive data collection effort was conducted by gathering gene–phenotype associations from a diverse set of 11 public and commercial sources. These sources include: Blueprint Genetics (https://blueprintgenetics.com/), Cegat (https://cegat.com/), Centogene (https://www.centogene.com/), ClinGen (https://clinicalgenome.org/), Fulgent Genetics (https://www.fulgentgenetics.com/), GeneDx (https://www.genedx.com/), Health in Code (https://healthincode.com/en/), Invitae (https://www.invitae.com/), PanelApp (https://panelapp.genomicsengland.co.uk/), Prevention genetics (https://www.preventiongenetics.com/), and Pronto diagnostics (https://www.prontodiagnostics.com/). Additionally, we incorporated data from the Human Phenotype Ontology [5] to enrich the phenotypic information associated with genes. Data were obtained exclusively from publicly available, online resources. Gene–phenotype association strength was categorized into tiers. Tiers were retrieved, where available, from each panel’s source specifications. Overall, three sources use three tiers to categorize gene–phenotype association (ClinGen, Health in Code, and PanelApp), one source uses two tiers (Invitae), and the rest use only a single list of genes per panel.

### Panel similarity calculation

To provide users with the capability to compare gene panels effectively, pairwise panel similarity was calculated for all possible panel pairs. Similarity calculation is based on a metric that takes into account the overlap of genes between two panels relative to the total number of genes in both panels. Mathematically, the similarity score (S) between two panels A and B is computed as follows:


$$S\left( {A,B} \right) = \frac{{\mid A\mathop \cap \nolimits^ B\mid }}{{\mid A\mathop \cup \nolimits^ B|}}$$

Here, |A ∩ B| represents the number of genes that are present in both panel A and panel B, and |A ∪ B| represents the total number of unique genes in both panels.

### GC content and genomic duplication data

To enhance the utility of the platform, we incorporate information corresponding to the sequencing complexity of each gene: specifically, we annotate gene regions with extreme guanine-cytosine (GC) content and putative genomic duplications. Genes in the designed panel are reviewed and those containing regions that are difficult to sequence and/or analyse are marked as such. The user may use this information and decide whether to issue a warning for panel genes that are difficult to analyse, recommend orthogonal validation, or even decide that given the expected complexity, it is not feasible to run the panel. GC content information was retrieved from the Genome in a Bottle stratifications dataset [6]. Putative genomic duplications [7] were retrieved from the University of Santa Cruz table browser data retrieval tool [8].

### Implementation

The platform was developed using R statistical software (version 4.0.2; R Foundation for Statistical Computing, Vienna, Austria) and the *shiny* R package [9].

## Functional features

**Figure 1.**
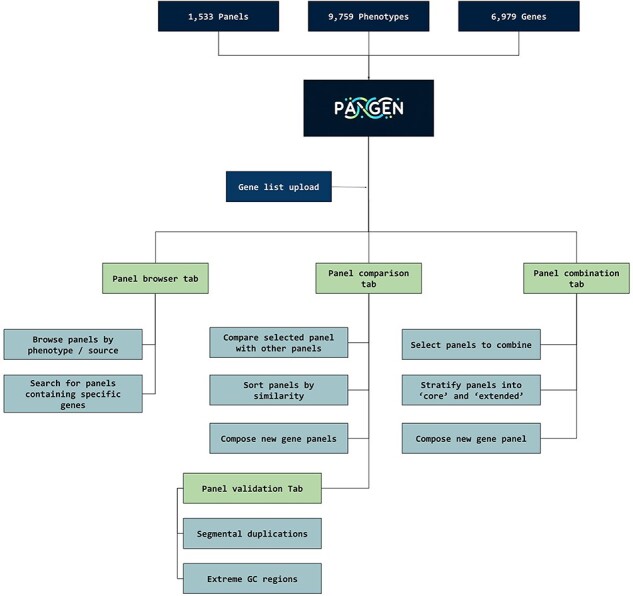
PANGEN facilitates targeted gene panel generation by allowing users to upload gene lists and interactively explore available panels by phenotype, identify similar panels, compare gene content, and generate personalized gene panels.

**Figure 2.**
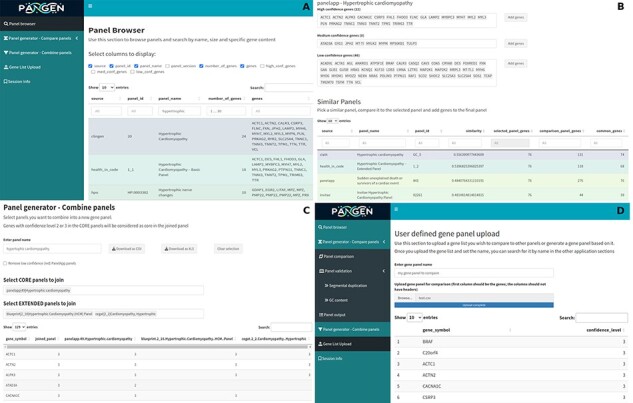
The online platform has several tabs the user select to perform various tasks. (A) Panel browser—search for specific gene panels of interest. (B) Panel comparison—compare a selected panel with other similar panels and generate a new panel based on user-defined combinations. (C) Panel combination—generate a two-tier panel based on a combination of selected ‘core’ and ‘extended’ panels, and (D) Gene list upload—upload a gene list that can then be compared and combined with other panels.

We have collected 6979 genes corresponding to 1533 panels and 9759 phenotypes. The platform was designed as an adaptable online interface and modified according to the feedback provided by our team of variant scientists. The platform offers various features for panel exploration and manipulation (Fig. 1): (i) Panel browser tab—users can search for specific gene panels of interest or browse through panels associated with particular clinical conditions or phenotypes or search for panels containing specific genes (Fig. 2A). (ii) Panel comparison tab—that compares a selected panel with all other collected panels (Fig. 2B). Panels can then be sorted by similarity, facilitating the addition of genes from similar panels and comparison with expert-opinion based panels (i.e. PanelApp). In this tab, the user can compose a new gene panel by adding genes from various sources. Panel similarity is a critical feature of our platform, allowing users to identify shared genes and commonalities between different panels. This information aids in the selection and customization of panels for specific research or clinical applications. (iii) Panel validation tabs—users can access duplication and GC content data on the platform. Genomic duplications and extreme GC content can complicate sequence alignment, leading to ambiguity in variant calling. To address this concern, we retrieve and present information on such putative genomic regions. These data aid users in assessing their gene panels and potential technical pitfalls in the downstream analysis. (iv) Panel combination tab, in which the user can select gene panels to combine (Fig. 2C). The platform allows users to stratify a generated panel into two categories: genes with strong phenotype associations (‘core’) and genes with weaker associations (‘extended’). This stratification helps clinicians and researchers to prioritize genes based on their perceived relevance to the phenotype under investigation. (v) Gene list upload tab, in which the user may upload a gene list that can then be compared and combined with other collected panels (Fig. 2D).

## Implementation

We utilized the platform and generated a set of 43 gene panels using the following procedure: (i) A PanelApp panel is selected according to a prespecified phenotype. (ii) All the genes in the PanelApp panel with either a ‘green’ or ‘amber’ tiers were assigned as ‘core’ panel genes. (iii) We then collect all related panels from other sources. Selection was based either on the name of the panels or their calculated similarity with the selected PanelApp panel. Every gene found in any of the other similar panels was assigned as ‘extended’ panel gene. These panels were uploaded to the platform and are currently available for comparison and review. Subsequently, during variant analysis, variants may be stratified and reported according to whether they affect ‘Core’ or ‘Extended’ genes by setting a lower reporting threshold for variants affecting ‘Core’ genes, as they are more likely to be associated with the phenotype in question. The compiled gene panels may be used for both targeted analysis and to narrow down the search space to the most phenotypically relevant genes in broader tests, such as WES/WGS.

The platform represents a unique, unified resource for gene panel exploration, comparison, and design that facilitates the combination of genes from different panels and from various sources to create panels tailored to specific research or clinical needs.

## Data Availability

The freely available web server can be accessed at: https://c-gc.shinyapps.io/PANGEN/.
